# Editorial

**Published:** 1839-10-19

**Authors:** 


### THE MEDICAL EXAMINER.

PHILADELPHIA, OCTOBER 19, 1839.

Much attention has been directed at New Orleans to the treatment of yellow fever by very large doses of the sulphate of quinine, given at the period of collapse, immediately at the commencement of the disease. This practice, which consists in giving large doses of quinine, say from a scruple to a drachm, has been resorted to, we believe, for two or three years past, with great advantage, to the diminution of the mortality of this disease, which, under ordinary methods of treatment, is singularly fatal.

We do not know at what exact date, or at whose suggestion, this practice was instituted. Most probably, like other modes of treatment, the idea has often been suggested, and successfully acted upon; but it is not long, at least, that the practice has been generally adopted, or that its success has been established upon tolerably certain grounds. In all cases of this kind, we are much more inclined to give the credit of a successful mode of treatment, or of the investigation of an obscure pathological problem, to those who first introduce it to the profession, by setting forth the testimony on which it rests, in a clear and unquestionable light, than to the physician who may have simply conceived the possibility of the connexion, but has let it remain in an useless and impracticable form.

The evidence respecting this new mode of treatment, to which we perceive an allusion in one of the late meetings of the Academy of Medicine of Paris, must be very complete to carry with it entire conviction. It is so easy to mistake the success of practice in epidemics, like yellow fever, which vary every year, and change their character sometimes in the course of a few weeks, that we should not entirely admit that any plan of treatment would be uniformly successful, until it had been tested by the epidemics of several successive years. Still, the experience of a single physician in a single year would furnish just grounds for adopting and testing a practice which may have been unusually successful in his hands, and may promise still better results when tried upon a larger scale.

We have thrown out these remarks more with the hope of eliciting some more definite information from our New Orleans correspondents than we now possess, than from any present knowledge we have respecting a mode of practice which, at least, promises more than any hitherto employed.

To put our readers in possession of what facts have come to light on this subject, we extract the following notice from a New Orleans paper:

*The Use of Quinine in Yellow Fever.*—The Lafayette Gazette mentions the salutary effect in cases of yellow fever, derived from the exhibition of the sulphate of quinine, in very large doses. Several facts coming under our immediate knowledge enable us to corroborate this statement, and to add that we believe the "quinine treatment," as it is termed, to be a most important and incalculably beneficial discovery.

The manner of employing the quinine in fever cases, which has been followed by such astonishing success, differs altogether from the mode in which that remedy is usually administered. The common practice of physicians has been to give it in small doses during the periods of remission. The new practice is based upon a different theory, and varies essentially from the old. When quinine is taken in large quantities, medical men have observed that it produces but a slight and inconsiderable stimulating effect, which is succeeded within a few hours by a powerful sedative impression, that is generally durable. With this view, the medicine is exhibited in one very large dose of from twenty to sixty or eighty grains in the very incipiency of the fever, while the morbid action appears to be in process of formation—that is, within six or eight hours, immediately after the appearance of the earliest symptoms. It is all important, if we rightly understand the theory of its use, that the quinine should be employed before local irritation or congestion has taken place, or, in other words, while the malady is confined to the nervous system, and the organization is as yet unimpaired. When taken under such circumstances, its first effects are, a very slight increase of the febrile symptoms; the pulse perhaps becomes quickened, the respiration more hurried, and the usual consequences of stimuli are present. This condition is, however, but transient, and is promptly followed by corresponding depression. All the more violent symptoms subside; the temperature of the surface is lowered; pain diminished; the pulse is gentle and subdued; the skin is covered with a healthy moisture: in short, the chain of morbid associations becomes broken—sleep is superinduced, from which the patient awakes refreshed, and substantially better; and within twenty-four or thirty-six hours is considered in a state of convalescence.

The treatment is, of course, not exclusively confined to the employment of quinine, though this is the chief remedial agent. The usual means of obviating tendencies to local irritation must be resorted to. The skilful practitioner will modify his curative measures according to the necessities of the case; cupping, leeching, the warm bath, and local applications, may be used as circumstances call for their employment. The quinine is administered in a single dose; the object of the physician is to bring about the sedative influence of the remedy, before any of the organs, as the head, stomach, &c., become specially affected by the disease. If it should fail to produce the anticipated effect, the case is too far advanced for a second trial, and it must be treated on general pathological principles. Let it, however, be remembered, that in thirty or forty cases which have been subjected to this novel curative method, not one has terminated fatally. The action of the quinine has been uniformly most salutary, operating like a charm, and dissipating the symptoms of the malady ere they become concentrated on particular organs.

We have been an eye witness of the excellent effects of the quinine treatment in several instances, and can with justice render a tribute to the zeal and talent displayed by some of the members of the profession in the employment of this remedy. We are not aware to whom the merit of the discovery belongs. Those physicians who have paid particular attention to its *modus operandi*, and have employed it in the largest number of cases, are Drs. Hunt, Beattie, Farrell and Mackay. These gentlemen concur in their views of the theory upon which the treatment is based, as well as in the unexceptionably advantageous results which accrue from its application.—*New Orleans Bee.*

---

A new edition of the complete works of Hippocrates, and of those which are erroneously ascribed to him in our printed collections, is now publishing at Paris, with a new and excellent French translation, by M. Littré. The edition will be comprised in seven volumes, one only of which has yet appeared. The first volume contains a small part only of the writings of Hippocrates; it is chiefly taken up with a learned and critical examination of the writings of the father of medicine, and of his medical opinions.

As a specimen of medical literature, this new edition of the works of Hippocrates is one of the most remarkable productions of the French medical press. Nor is it the only work of the ancient classical writers on medicine which has received this care; a similar edition of the works of Aretäus was lately published, and there are found numerous late publications exclusively devoted to historical medicine, especially the large dictionary of M. Dezeimeris.

These publications show that the modern tendency to examine questions of pathology by a means of investigation unknown to the ancients, does not bring about an utter disregard for the writings of the fathers of medicine. On the contrary, the care which is now taken to place them in a condition for easy and frequent perusal, shows that the discovery of a new mode of eliciting truth, can never cast discredit upon facts which are clothed in a venerable and oddly-fashioned garment.

Evidences of a strong and renewed attention to the older writers are becoming daily more general, and we hope that the publication of the works of many of our predecessors will be promoted by the medical press of America. The cheap and useful collections periodically published in this city, afford singular facilities for such an undertaking; and if they have not yet exhausted the stock of the more recent publications, the time must necessarily soon arrive when the supply of European works of a high character will be inadequate to the demands of a press which compresses a volume into the size of a pamphlet, and, in a few months, absorbs the accumulated literature (good and indifferent) of as many years.

